# Development and validation of a predictive nomogram for frailty based on thyroid function in older adults

**DOI:** 10.1007/s41999-025-01247-3

**Published:** 2025-06-17

**Authors:** Xiaotian Shi, Huayu Yang, Shan Wang, Yifan Yang, Yuanyuan Li, Guoze Dou, Qing Ma

**Affiliations:** 1 Present Address: Department of Geriatrics, Beijing Friendship Hospital, Capital Medical University, No. 95 Yong’an Road, Beijing, 100050 People’s Republic of China; 2 School of General Practice and Continuing Education, Capital Medical University, Beijing, People’s Republic of China

**Keywords:** Thyroid hormone, Frailty, Nomogram, Prediction model

## Abstract

**Introduction:**

This study aimed to develop and validate a frailty prediction model incorporating thyroid hormone levels in older adults with normal thyroid function.

**Methods:**

A total of 767 participants aged ≥ 65 years (566 males, 73.8%) were enrolled between December 2019 and December 2023. Participants were randomly assigned to training (*n* = 546) and internal validation (*n* = 235) sets; an external validation cohort was recruited between December 2023 and December 2024. Frailty was assessed using the FRAIL scale. Predictors were selected through LASSO and logistic regression analyses, and a nomogram was constructed for clinical application.

**Results:**

A total of 767 patients were included, with a median age of 81 years. Of these, 205 individuals (26.7%) were classified into the frailty group. The restricted cubic spline plot showed a negative correlation between the FT3/FT4 and frailty after adjusting for multiple confounding factors. Five predictors, age, polypharmacy, MNA.SF score, grip, and FT3/FT4 were incorporated to establish the model. The model performed well achieving AUC values of 0.92 and 0.80 in the development and external validation datasets, respectively. The Calibration curves and DCA results suggested that the model possesses satisfactory predictive performance.

**Conclusions:**

We developed a clinically applicable model to predict frailty in older adults with normal thyroid function, highlighting the relevance of thyroid hormone status in frailty risk assessment.

**Supplementary Information:**

The online version contains supplementary material available at 10.1007/s41999-025-01247-3.

## Key summary points

**Aim:**

Recent evidence links thyroid hormone profiles to age-related conditions. This study explored the association between thyroid function and frailty in older adults with normal thyroid levels, and aimed to develop and validate a frailty prediction model incorporating thyroid function.

**Findings:**

A lower FT3/FT4 ratio was associated with increased frailty risk. A predictive model including age, polypharmacy, MNA-SF score, grip strength, and FT3/FT4 ratio showed good performance in identifying frailty.

**Message:**

We propose a practical model for predicting frailty in older adults with normal thyroid function, highlighting the FT3/FT4 ratio as a useful indicator.

**Supplementary Information:**

The online version contains supplementary material available at 10.1007/s41999-025-01247-3.

## Introduction

Frailty is characterized by a multifaceted decline in physiologic function across multiple systems, resulting in heightened vulnerability to stressors [1, 2]. It is associated with adverse outcomes and increased healthcare expenditures [3, 4]. The prevalence of frailty varies widely across studies, ranging from 4 to 59%, owing to the lack of standardization in definitions and measurement methods [5]. However, a simple biochemical marker of frailty has yet to be identified.

Aging is characterized by the gradual decline in the function of multiple organ systems, accompanied by impaired tissue maintenance and repair. As a key endocrine organ, the thyroid plays a pivotal role in development, growth, and energy metabolism. Recent studies have highlighted the significant impact of thyroid hormones on tissue homeostasis and repair processes[6]. Studies investigating the correlation between thyroid dysfunction and adverse outcomes have found associations between thyroid status and age-related diseases such as diabetes, coronary heart disease, anemia, and heart failure[7–9]. However, these findings exhibit inconsistency across various cohort analyses. In some cases, the relationship between thyroid status and prognosis appears to be linear, whereas in other cases, a U-shaped association is observed [10].

The pathogenesis of frailty is complex, encompassing factors such as chronic inflammation, oxidative stress, neuroendocrine dysregulation, and immune dysfunction [11, 12]. Thyroid dysfunction has wide-ranging effects on various aspects of health, including glucose and lipid metabolism, musculoskeletal function, cognition, and the cardiopulmonary system. Several studies have demonstrated that abnormal thyroid function is associated with an increased risk of frailty among thyroid hormone indicators. Both free thyroxine (FT4) and thyroid-stimulating hormone (TSH) have been shown to correlate with frailty [13, 14]. However, only a limited number of studies have evaluated the potential relationship between thyroid function and frailty in individuals with normal thyroid function. Due to the use of varying indices of thyroid hormone levels and differing frailty assessment methods, these studies have yielded inconsistent results [15, 16]. Although frailty prediction models have been developed, no study has yet incorporated thyroid function into such models. Therefore, this study aims to investigate the correlation between thyroid function and frailty in older individuals with normal thyroid function, and to develop and validate a new frailty prediction model that includes thyroid function.

## Methods

### Patients and study design

We selected the data for model development from December 2019 to August 2023. The data were obtained from patients at Beijing Friendship Medical Center, affiliated with Beijing Friendship Hospital and Capital Medical University.

The inclusion criteria were as follows: [a] adults aged 65 and above who have completed geriatric assessment; [b] subjects who agree to sign informed consent forms. The exclusion criteria were as follows: [a] patients who have previously been diagnosed with thyroid disease; [b] participants who have been on chronic treatment with drugs known to affect thyroid function, such as thyroid hormone preparations, methimazole, propylthiouracil, amiodarone, and lithium. [c] Patients who have undergone thyroid surgery or radiation iodine therapy. In addition, we used the data for external validation from December 2023 to December 2024 at Beijing Friendship Medical Center affiliated with Beijing Friendship Hospital and Capital Medical University. The inclusion and exclusion criteria were consistent with the development cohort.

This study was registered at www.chictr.org.cn. (ChiCTR2400080562) and was approved by the Clinical Analysis Commission of the Beijing Friendship Hospital, Capital Medical University (Project number: 2023-P2-303–02).

### Clinical evaluations and laboratory tests

The socio-demographic characteristics included gender, age, body mass index (BMI), educational level, smoking status, and current medication usage. Leukocytes, red blood cells, urea nitrogen, thyroid hormones, and other relevant factors were measured in all participants. Serum thyroid function tests were performed using the Roche Cobas e801 electrochemiluminescence immunoassay system (Roche Diagnostics, Switzerland). The reference ranges for normal values of thyroid hormones displayed below: TSH: 0.35–4.94uIU/mL; total triiodothyronine (T3): 0.89–2.45 nmol/L; total thyroxine (T4): 62.68–150.84 nmol/L; free triiodothyronine (FT3): 2.63–5.71 pmol/L; and free thyroxine (FT4): 12.00–22.00 pmol/L. Serum samples were analyzed at the Beijing Friendship Hospital Health Care Center Laboratory, using a standardized and certified system.

### Comprehensive geriatric assessment

All assessors had backgrounds in medicine or nursing and completed standardized geriatric training prior to conducting assessments. The FRAIL score, which evaluates fatigue, muscle resistance, ambulation, illness, and weight loss, ranges from 0 to 5. It assesses frailty based on responses to five yes-or-no questions. Participants were classified as robust (0 points), pre-frail (1–2 points), or frail (3–5 points). Mini Nutritional Assessment-Short Form (MNA.SF) is a concise tool to measure nutritional status. This scale has been validated in the Chinese population [17].In addition, nutritional status was assessed using the Geriatric Nutritional Risk Index (GNRI). The GNRI was calculated using the following formula: GNRI = 1.489 × serum albumin (g/L) + 41.7 × (actual body weight / ideal body weight), where the ideal weight was estimated using the Lorentz formula. If actual weight exceeded ideal weight, the ratio was set to 1. GNRI values were categorized into four groups: no risk (≥ 98), low risk (92–97), moderate risk (82–91), and severe risk (< 82) [18].

Grip strength was assessed using CAMRY spring handgrip dynamometers, with the elbow fully extended in the standing position. If the participant was unable to stand, grip strength was measured in the sitting position. Participants were instructed to contract both hands with maximum force and undergo at least two tests to select the maximum reading.

### Statistical analysis

Continuous variables were analyzed using either the Mann–Whitney U test or independent t test, while differences in categorical variables were assessed through the chi-squared test or Fisher’s exact test. The study examined the relationship between the FT3/FT4 ratio and frailty by adjusting for potential confounding factors through a smooth curve analysis. To comprehensively evaluate the influence of nutritional status on the association between the FT3/FT4 ratio and frailty, spearman correlation analyses were first performed to explore the relationship between FT3/FT4 and nutritional status indicators (MNA-SF and GNRI). Then, binary logistic regression was used to examine the independent association between FT3/FT4 and frailty after adjusting for potential confounders, including nutritional status indicators. To further determine whether the association between FT3/FT4 and frailty was modified by nutritional status, interaction terms (FT3/FT4 × MNA-SF; FT3/FT4 × GNRI) were created and included in the regression models. In addition, stratified analyses were conducted according to MNA-SF categories and GNRI-defined nutritional risk groups to assess potential effect modification. Cross-tabulation was performed, and Cohen’s kappa coefficient was calculated to assess the level of agreement between MNA-SF and GNRI classification.

Data were randomly divided into training (*n* = 546) and validation (*n* = 235) sets, according to a ratio of 7:3 [19]. The least absolute shrinkage and selection operator (LASSO) regression analysis was used to develop and validate the model. Significant variables identified in the univariate analysis were included in the construction of a predictive model using multivariable logistic regression. An ROC curve was generated to assess the predictive performance of the model. Calibration was assessed using a calibration curve (bootstrap, *n* = 1000) and the Hosmer–Lemeshow test. Decision curve analysis (DCA) was employed to assess the clinical utility of the prediction model. In addition, a nomogram was developed to provide a visual representation of the predictive model. R software (version 4.2.2) was used for all analyses in this study. Two-tailed tests were performed, with *P* < 0.05 considered statistically significant.

## Results

### Patient characteristics

The development dataset comprised 767 participants, of whom 205 older adults (26.7%) were identified as frail. There were 566 male participants (73.8%), with a median age of 81 years. In the external validation dataset, a final analysis was conducted on 190 participants, revealing 58 older adults with frailty. Of the 119 male cases (62.6%), the median age was 80 years. Table 1 shows the baseline characteristics of the patients divided into four groups according to quartiles of FT3/FT4 levels. There were significant differences in age, polypharmacy, smoking history, and BMI, while there were no significant differences in gender between groups. Significant differences were found in the grip strength, MNA.SF and frail scale (all *P* < 0.001, Table 1). A comparison of the four groups showed significant differences in WBC, RBC, RGB, ALB, ALT, BUN, UA, Cr, CHOL, LDL, TG, T3, FT3, FT4, TSH,

**Table 1:** Baseline characteristics of participants based on quartiles of FT3/FT4

Variables	Q1 (*n* = 191)	Q2 (*n* = 193)	Q3 (*n* = 191)	Q4 (*n* = 192)	*P*
Age(years)	86.0 (80.0, 90.5)	82.0 (74.0, 88.0)	78.0 (69.0, 84.5)	73.0 (67.7, 82.0)	< 0.001
Gender					0.495
Male	144 (75.3%)	148 (76.6%)	139 (72.7%)	135 (70.3%)	
Female	47 (24.6%)	45 (23.3%)	52 (27.2%)	57 (29.6%)	
Polypharmacy	116 (60.7%)	100 (51.8%)	89 (46.6%)	77 (40.1%)	< 0.001
Smoking	14 (7.3%)	23 (11.9%)	24 (12.5%)	32 (16.6%)	0.049
BMI (Kg/m^2^)	22.8 (20.1, 25.3)	24.4 (22.2, 26.5)	24.2(21.9, 26.3)	24.3 (22.1, 27.0)	< 0.001
Grip (Kg)	20.2 (14.7, 26.7)	22.9 (17.7, 30.4)	27.4 (22.7, 33.6)	29.5 (23.2, 35.6)	< 0.001
FRAIL group					< 0.001
Non-frail	88 (46.0%)	132 (68.3%)	154 (80.6%)	187 (97.4%)	
Frail	103 (53.9%)	61 (31.6%)	37 (19.3%)	5 (2.6%)	
MNA.SF (Score)	11.0 (9.0, 13.0)	13.0 (11.0, 14.0)	13.0 (11.0, 14.0)	13.0 (12.0, 14.0)	< 0.001
HBA1c (%)	5.96 (5.50, 6.85)	5.94 (5.54, 6.84)	5.90 (5.54, 6.63)	5.96 (5.61, 6.40)	0.968
WBC (× 10^12^/L)	6.74 (5.46, 8.84)	6.38 (5.14, 7.63)	6.21 (5.12, 7.84)	6.15 (5.17, 7.34)	0.002
RBC (× 10^12^/L)	3.99 (3.50, 4.45)	4.23 (3.80, 4.60)	4.39 (4.08, 4.81)	4.54 (4.21, 4.86)	< 0.001
HGB (g/L)	122.0(109.0, 137.0)	132.0 (120.0, 142.0)	138.0 (127.0, 148.0)	141.0 (131.0, 152.0)	< 0.001
ALB(g/L)	33.7 (31.1, 37.0)	37.1 (34.3, 41.0)	40.4 (36.7, 43.25)	41.6 (38.7, 44.9)	< 0.001
ALT(U/L)	14.0 (10.0, 23.0)	15.00 (11.0, 21.0)	16.00 (12.0, 21.0)	17.00 (13.0, 26.0)	0.002
AST (U/L)	20.0 (16.0, 27.0)	20.0 (17.0, 25.0)	22.0 (18.0, 25.0)	21.5 (19.0, 26.0)	0.080
BUN (mmol/L)	5.82 (4.71, 8.27)	5.44 (4.28, 6.67)	5.52 (4.64, 6.44)	5.20 (4.38, 6.35)	0.005
UA (umol/L)	321.60 (247.00, 399.70)	325.40 (266.50, 374.60)	341.20 (282.00, 401.05)	338.10 (295.88, 394.10)	0.015
Cr (umol/L)	72.0 (63.4, 79.4)	63.6 (57.5, 72.0)	62.4 (56.1, 67.7)	58.6 (53.0, 63.8)	< 0.001
GLU (mmol/L)	5.67 (4.80, 6.75)	5.35 (4.73, 6.68)	5.58 (4.96, 6.95)	5.74 (5.08, 6.60)	0.308
CHOL(mmol/L)	3.78 (3.02, 4.39)	3.83 (3.17, 4.66)	4.11 (3.54, 4.91)	4.45 (3.78, 5.21)	< 0.001
TG(mmol/L)	0.98 (0.76, 1.19)	1.09 (0.80, 1.38)	1.21 (0.90, 1.70)	1.28 (1.01, 1.76)	< 0.001
HDL(mmol/L)	1.07 (0.84, 1.26)	1.06 (0.84, 1.26)	1.09 (0.89, 1.29)	1.08 (0.93, 1.32)	0.06
LDL(mmol/L)	2.16 (1.67, 2.60)	2.22 (1.82, 2.86)	2.47 (2.00, 2.99)	2.57 (2.13, 3.13)	< 0.001
T3(nmol/L)	1.03 (0.86, 1.25)	1.27 (1.08, 1.46)	1.45 (1.32, 1.65)	1.58 (1.38, 1.76)	< 0.001
T4(nmol/L)	111.15 (93.17, 129.12)	112.46 (94.66, 130.68)	113.57 (99.15, 127.07)	108.88 (98.01, 123.31)	0.232
FT3(pmol/L)	3.71 (3.37, 4.07)	4.35 (3.97, 4.66)	4.73 (4.44, 5.07)	5.18 (4.78, 5.56)	< 0.001
FT4(pmol/L)	13.97 (12.59, 15.12)	12.25 (11.25, 13.11)	11.33 (10.46, 12.16)	10.00 (9.40, 10.84)	< 0.001
TSH (uIU/ml)	1.46 (0.95, 2.21)	1.84 (1.13, 2.79)	1.88 (1.17, 3.24)	2.42 (1.50, 3.44)	< 0.001
FT3FT4ratio	0.28 (0.25, 0.30)	0.35 (0.34, 0.37)	0.42 (0.40, 0.44)	0.50 (0.48, 0.54)	< 0.001

Continuous variables are expressed as median (interquartile range) and categorical data using number (percentage)

### The correlation between FT3/FT4 and frailty

**Fig. 1:**
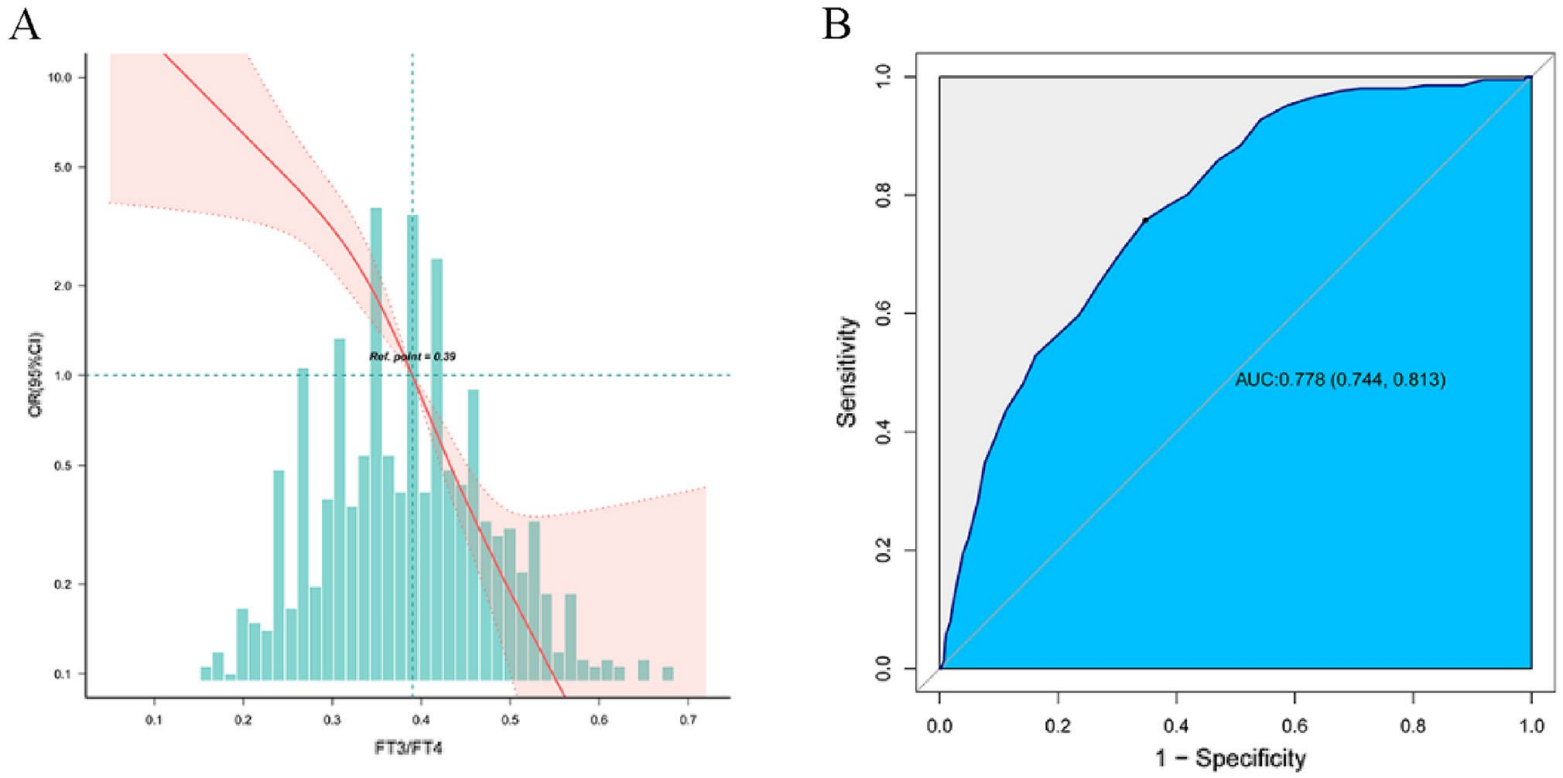
A. Restricted cubic spline (RCS) of the association between the FT3/FT4 and frailty. B.ROC curve of FT3/FT4 prective frailty

The restricted cubic spline plot showed a significantly negative correlation between the FT3/FT4 and frailty (Fig. 1A). The unadjusted model showed a negative correlation between FT3/FT4 and frailty, which remained significant after adjusting for age and gender. Fully model corrected gender, age, BMI, smoking, MNA.SF, grip, HbA1c, WBC, RBC, HGB, CHOL, TG, HDL, LDL, UA, BUN, Cr, AST, ALT, T3, T4, FT3, FT4, and TSH and showed that the FT3/FT4 had a negative correlation with the risk of frailty. Compared with the Q1 group, the FT3/FT4 in Q4 group had a significantly lower risk of frailty (OR = 0.04, 95% CI: 0.01–0.13) (Table 2). The ROC curve showed an AUC of 0.778 (95% CI: 0.744–0.813), with a sensitivity of 75.7% and specificity of 65.2% (Fig. 1B).

**Table 2:** Relationship between FT3/FT4 and frailty in different models

Variable	Crude model (OR, 95% CI, *p*)	Minimally adjusted model (OR, 95% CI, *p*)	Fully adjusted model (OR, 95% CI, *p*)
FT3/FT4 (quartile)			
Q1	Reference	Reference	Reference
Q2	0.39 (0.26, 0.60) < 0.001	0.51 (0.32, 0.84) 0.008	0.94 (0.50, 1.75) > 0.05
Q3	0.21 (0.13, 0.32) < 0.001	0.41 (0.24, 0.69) < 0.001	0.97 (0.49, 1.93) > 0.05
Q4	0.02 (0.01, 0.06) < 0.001	0.04 (0.02, 0.11) < 0.001	0.04 (0.01, 0.13) < 0.001
*p* for trend	< 0.001	< 0.001	< 0.001

Crude model: we did not adjust other covariants

Minimally adjusted model: adjusted age and gender

Fully adjusted model: adjusted age, gender, BMI, smoking, MNA.SF, grip,HbA1c,WBC,RBC, HGB,CHOL,TG, HDL, LDL, UA, BUN, Cr, AST,ALT,T3,T4,FT3,FT4,TSH

### Association between FT3/FT4 and frailty independent of nutritional status

To examine whether the association between FT3/FT4 and frailty is independent of nutritional status, we first analyzed the correlation between FT3/FT4 and MNA-SF scores. A positive correlation was observed (*r* = 0.321, *P* < 0.001), suggesting that a higher FT3/FT4 ratio is associated with better nutritional status. In multivariable logistic regression models adjusting for MNA-SF, FT3/FT4 remained significantly associated with frailty (OR = 0.04, 95% CI: 0.01–0.13), indicating that this association is independent of nutritional status. Interaction analysis showed no significant interaction between FT3/FT4 and MNA-SF (*P* > 0.05), suggesting the association between FT3/FT4 and frailty was consistent across nutritional levels. For stratified analysis, participants were categorized into normal nutrition (*n* = 523, 68.2%), at risk of malnutrition (*n* = 198, 25.8%), and malnutrition (*n* = 46, 6.0%) based on MNA-SF. Due to the small sample size in the malnourished group and non-significant interaction between the two at-risk groups (*P* > 0.05), these were combined into a single malnutrition group (Table S1). The association between FT3/FT4 and frailty remained consistent in both the normal and malnourished groups.

Following the analysis using MNA-SF, we further examined the association between FT3/FT4 and frailty using GNRI as an alternative nutritional assessment tool. Among the study population, 352 participants (45.9%) were classified as having no nutritional risk, 209 (27.2%) as mild risk, 162 (21.1%) as moderate risk, and 44 (5.7%) as severe risk according to the GNRI. Correlation analysis showed a significant negative correlation between FT3/FT4 and GNRI scores (*r* = – 0.508, *P* < 0.001), indicating that a lower FT3/FT4 ratio was associated with worse nutritional status. Independent association analysis revealed that, even after adjusting for GNRI in multivariate models, FT3/FT4 remained significantly associated with frailty, suggesting that its association with frailty is independent of nutritional status (Table S2). Interaction analysis found no significant interaction between FT3/FT4 and GNRI (*P* > 0.05), indicating that the effect of FT3/FT4 on frailty did not vary significantly across GNRI-defined risk levels. Stratified logistic regression showed that the association between FT3/FT4 ratio and frailty remained statistically significant in participants with no nutritional risk and mild nutritional risk, but this association was not observed in those with moderate-to-severe nutritional risk (Table S3).

## Agreement analysis between gnri and mna-sf

To assess the consistency between different nutritional assessment tools and to explore the potential reasons for discrepancies observed in the stratified analysis of FT3/FT4 and frailty, we conducted an agreement analysis between the GNRI and MNA-SF classifications. Based on the MNA-SF, 523 participants (68.2%) were classified as having normal nutritional status, and 244 (31.8%) as being at risk or malnourished. According to the GNRI, 352 participants (45.9%) were in the no-risk group, and 415 (54.1%) were at nutritional risk. The cross-tabulation of MNA-SF and GNRI revealed a significant association between the two nutritional assessment tools (*χ*^2^ = 101.55, *P* < 0.001). The Cohen’s kappa coefficient was 0.32, indicating fair agreement between the two tools in identifying nutritional risk. This suggests that although the tools overlap to some extent, they may capture different dimensions of nutritional status.

### Predictors selection and model development

**Fig. 2:**
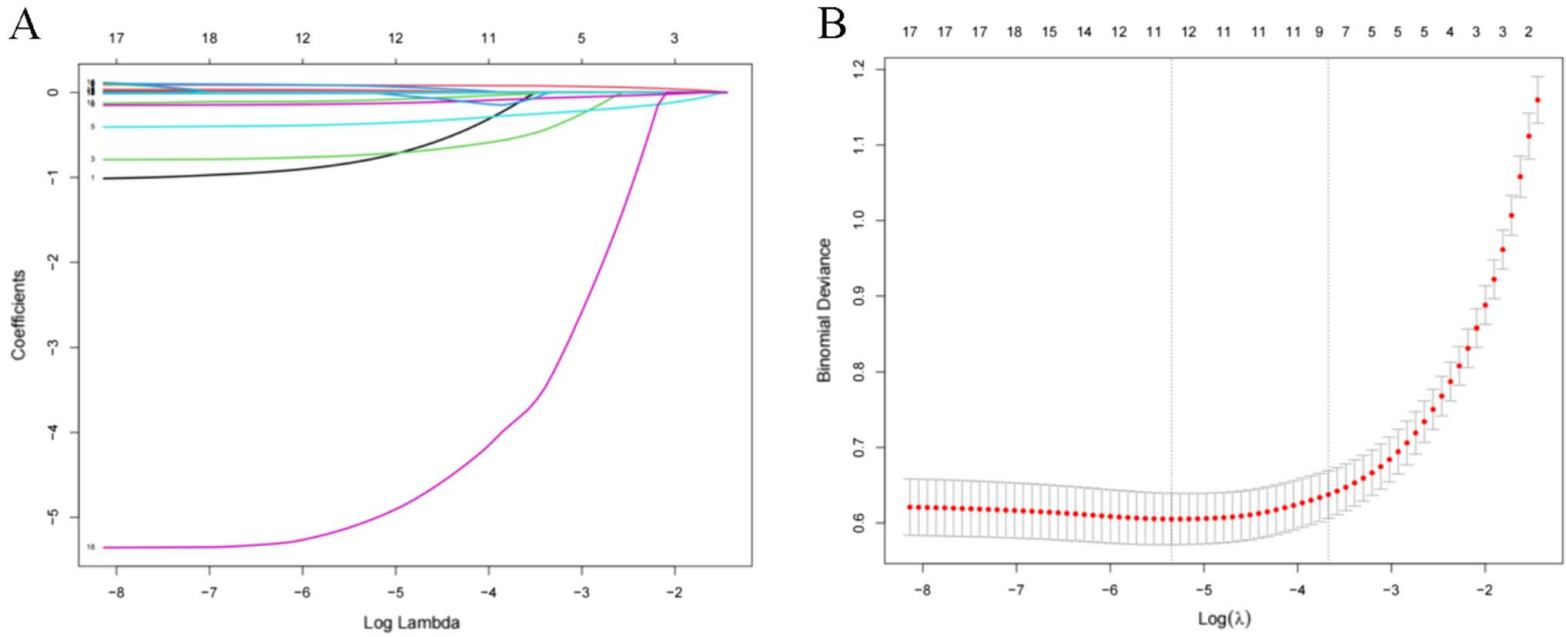
**A** Coefficient curves for clinical features. **B** Lasso regression tenfold cross-validation to select the most appropriate clinical features. A virtual vertical line at the optimal value was drawn using one SE of minimum criterion (the 1-SE criterion)

**Fig. 3:**
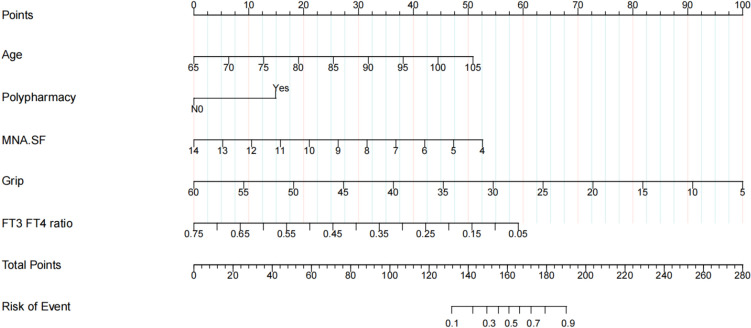
The nomogram including age, MNA.SF, grip, polypharmacy and FT3/FT4 was plotted to represent the predicting model

Predictors were further screened using LASSO regression analysis, with tenfold cross-validation with a random seed value of 110. Non-zero coefficients identified through this process were considered potential predictors (Fig. 2A, B). The meaningful factors selected through lasso regression analysis were incorporated into the logistic regression model. Multivariable analysis revealed that age (OR = 1.08, 95% CI: 1.04–1.12), polypharmacy (OR = 2.27, 95% CI: 1.23–4.22), grip strength (OR = 0.92, 95% CI: 0.89–0.96), MNA.SF (OR = 0.65, 95% CI; 0.57–0.74) and FT3/FT4 (OR = 0.02, 95% CI: 0.01–0.57) were independent risk factors for frailty. The predictive model was developed and presented as a nomogram (Fig. 3). Each variable was assigned a score on the x-axis. Summing these scores yielded the total score, which indicated the probability of frailty in older individuals. Higher total scores corresponded to a greater likelihood of frailty.

The Variance Inflation Factor (VIF) was used to assess multicollinearity among the variables in the logistic regression model, and the results showed that the VIF values for all variables was less than 5, suggesting an absence of significant multicollinearity.

### Evaluation of the prediction model

The predictive model demonstrated excellent performance, achieving an AUC of 0.92 in both the training and validation sets (Figure S1 A and B). The Hosmer–Lemeshow test demonstrated a good fit for the model in both the training set (*x*^2^ = 5.96, *P* = 0.65 > 0.05) and the validation set (*x*^2^ = 4.30, *P* = 0.82 > 0.05). Figure S1 C and D present the calibration plots for both the training and validation sets, illustrate a strong alignment between the predicted and observed probabilities of frailty in both the training set and the validation set. The higher degree of agreement between the predicted probabilities and the actual outcomes (*P* > 0.05) further confirmed the reliability and accuracy of the nomogram model’s predictive performance. The clinical utility of the nomogram model was evaluated using the DCA method. The x-axis, ranged from 0 to 1.0, representing the threshold probability for frailty; the y-axis represented the net benefit; the black line indicated the scenario where no patients were predicted to have frailty; the gray dashed line represented the scenario where all patients were predicted to have frailty; and the blue line indicated the net benefit of the nomogram model. The DCA curves showed that the net benefits of the nomogram model for both the training (Figure S1 E) and validation (Figure S1 F) sets consistently exceeded the black and gray lines, suggesting superior clinical applicability of this predictive model. These results indicate that the nomogram possesses significant discriminatory capability and predictive value.

### External model validation

In the external validation, the model performed better, achieving a discrimination AUC of 0.80 (95% CI 0.719–0.891). External validation indicated that the discrimination AUC, calibration *χ*^2^, and calibration slope means were acceptable, demonstrating good overall consistency of our equations (Figure S2).

## Discussion

Our findings revealed that FT3/FT4 was associated with frailty score in older people with normal thyroid function. In addition, we developed a predictive model for older patients with frailty based on age, polypharmacy, grip strength, MNA.SF and FT3/FT4.

The thyroid gland plays a crucial role in the process of aging, particularly in age-related diseases. A Korean study has indicated that a low FT3/FT4 ratio was a reliable indicator of low muscle mass in older people with normal thyroid function [20]. A cross-sectional study of 1,868 participants found that among middle-aged and older individuals with normal thyroid function, a reduced FT3/FT4 ratio is associated with decreased muscle mass [21]. Ostan R et al. also found that FT3/FT4 ratio significantly decreased with age, and lower FT3/FT4 levels were associated with functional impairment and increased the risk of mortality [22]. Frailty is fundamentally associated with sarcopenia; therefore, we investigated the relationship between the FT3/FT4 and frailty. Our findings imply that a high FT3/FT4 was substantially related with a lower risk of frailty in the older people with normal thyroid function in China. Another study identified the FT3/FT4 as the most robust independent indicator of frailty among all thyroid hormone indicators in the older adults [23]. PASQUALETTI et al. demonstrated a significant correlation between a decreased FT3/FT4 ratio and poor scores on the Multidimensional Geriatric Assessment and Multi Prognostic Index in hospitalized patients. Moreover, the FT3/FT4 independently predicts survival [24]. A cohort study revealed a significant negative correlation between the FT3/FT4 ratio and frailty. In addition, it was observed that an increase in the FT3/FT4 is associated with a gradual decrease in mortality risk [15].

Within this specific population, variations in the FT3/FT4 ratio were found to be associated with frailty. This finding supports the potential role of the FT3/FT4 ratio as a biomarker of functional status, although causality cannot be inferred. Further research is warranted to explore the underlying molecular mechanisms. Existing research has demonstrated that thyroid hormones are key regulators of the body’s capacity to maintain overall organ function and are critical for sustaining physical activity and physiologic processes [25]. FT3 is the biologically active form of thyroid hormone. During the aging process, deiodinase activity may initially compensate for declining thyroid function by enhancing T4-to-T3 conversion, thereby maintaining circulating FT3 levels. However, this compensatory capacity becomes progressively depleted over time, ultimately leading to a progressive decline in serum FT3 concentrations [26]. The FT3/FT4 ratio is regarded as an indirect marker of thyroid deiodination disorders. Compared to FT3 or FT4 alone, the FT3/FT4 ratio serves as a surrogate indicator for assessing the extent of peripheral thyroid hormone deiodination and deiodinase activity. It reflects subtle metabolic alterations in thyroid hormones and exhibits a stronger correlation with prognosis [27]. FT3/FT4 ratio may serve as an indicator of 5′-deiodinase (D1) activity. Individuals with a relatively high FT3/FT4 ratio are likely able to preserve D1 activity. In these individuals, the age-related decline in serum T3 levels may be counterbalanced by a compensatory increase in D1 activity. This adaptive mechanism, aimed at maintaining adequate local production of active T3, could facilitate the preservation of thyroid hormone signaling, as recently suggested with the concept of “thyroid hormone profile” [28]. Nonthyroidal illness syndrome (NTIS) is characterized not only by peripheral alterations in deiodinase activity but also by central suppression of the hypothalamic-pituitary-thyroid (HPT) axis, including decreased secretion of thyrotropin-releasing hormone (TRH). Typically, the earliest hormonal change observed is a decline in FT3 levels; however, as the severity of illness increases, reductions in TSH and FT4 levels also occur [29]. This complex endocrine adaptation reflects the body’s response to systemic illness and should be carefully considered when interpreting thyroid hormone alterations in the context of frailty. Therefore, changes in thyroid hormones in frail individuals may not only indicate peripheral metabolic dysregulation but also central neuroendocrine involvement.

One possible explanation for the lack of association between FT3/FT4 and frailty in individuals with moderate-to-severe nutritional risk may be that, in these patients, malnutrition itself has a predominant influence on the development of frailty, potentially overshadowing the contribution of hormonal parameters [30]. Moreover, it is also possible that low FT3/FT4 in malnourished individuals reflects a non-thyroidal illness syndrome, which may weaken its predictive power for frailty [31]. In contrast, among individuals with relatively preserved nutritional status, the FT3/FT4 ratio may better reflect subtle endocrine or metabolic disturbances associated with the frailty phenotype. In this study, the agreement between GNRI and MNA-SF in classifying nutritional risk was fair (*κ* = 0.32). This finding implies that while the two tools are significantly associated, they assess nutritional status from different perspectives. GNRI is based on serum albumin and weight and may reflect more chronic or physiologic aspects of malnutrition. In contrast, MNA-SF includes both objective and subjective components, such as appetite, mobility, and psychologic stress, potentially identifying earlier or more functional declines. This difference may account for the observed inconsistency in the stratified analysis of FT3/FT4 and frailty. FT3/FT4 remained significantly associated with frailty in all strata defined by MNA-SF, but the association was attenuated or absent in moderate-to-severe GNRI-defined risk groups. These results suggest that thyroid hormone ratios may better reflect frailty risk in the context of early or functional nutritional deficits, but may lose predictive power in the presence of more advanced physiologic malnutrition. Therefore, caution is warranted when interpreting FT3/FT4 in relation to frailty, and the selection of nutritional assessment tools should consider their respective underlying constructs.

It is currently posited that inadequate peripheral conversion of thyroxine represents a systemic adaptive response to tissue damage, serving as an indicator of disease severity. Furthermore, the decrement in thyroid hormone deiodination is associated with imbalances in oxidative stress and systemic inflammatory response processes [32]. The pathologic and physiologic mechanisms underlying frailty are intricate, with chronic inflammation and oxidative stress considered as pivotal factors in its development [33, 34]. Alterations in thyroid hormone levels may also be associated with chronic inflammation during aging. Previous studies have confirmed that frailty was associated with systemic inflammation as evidenced by elevated levels of Tumor Necrosis Factor-alpha (TNF-α) and Interleukin-6 (IL-6) [35]. Future studies could explore the correlation between changes in thyroid hormone levels and inflammatory factors. Recent evidence further suggests that the age-related decline in T3 levels should no longer be viewed as a simple physiologic aging process but rather as a potential marker of underlying pathologic conditions, including chronic inflammation and chronic diseases. Pro-inflammatory cytokines, including tumor necrosis factor-beta (TNF-β) and IL-6, have been shown to inhibit type I deiodinase activity, leading to reduced FT3 levels and concomitant increases in reverse T3 (rT3) levels [36]. These changes in thyroid hormone metabolism may contribute to the development of the frailty phenotype, indicating that low T3 status may reflect a state of chronic low-grade inflammation. Future studies could further explore the correlation between thyroid hormone alterations and inflammatory mediators.

The prevalence of frailty increases with age, imposing a significant burden on public health systems. Therefore, developing a predictive model to identify potential factors influencing the occurrence of frailty is essential for the clinical management of the older adults. The model incorporates five key factors: age-related comorbidities, polypharmacy, grip strength, nutrition, and FT3 to FT4 ratio. Age-related changes are associated with a decline in biologic function and increased vulnerability, which contribute to the onset of frailty [4]. There is a complex interrelationship between frailty and polypharmacy. As individuals age, their metabolic capacity declines, leading to altered pharmacokinetics. This leads to prolonged drug retention in the body, and an increased risk of adverse reactions, which can accelerate the onset and progression of frailty [37]. In addition, nutrition plays a critical role in frailty. Malnutrition leads to a decrease in muscle strength and fatigue, which interact with each other and perpetuate a vicious cycle of frailty-malnutrition [38]. Grip strength is considered as a crucial component of the fried phenotype and serves as a key assessment indicator for sarcopenia. Sarcopenia, a systemic skeletal muscle disorder characterized by loss of muscle mass and function, represents a significant contributor to frailty [39]. Research has shown that grip strength can be utilized in conjunction with other indicators to screen older people for various health conditions, and served as a crucial metric for evaluating physical function [40]. Our study also identified that lower FT3 to FT4 ratio was an independent risk factor for frailty in older adults, with a lower ratio indicating an increased risk of frailty. However, previous predictive models focusing on frailty have paid limited attention to thyroid Function. In this study, the nomogram model is the first to identify the FT3/FT4 ratio as a potential risk factor for predicting frailty in older adults. This finding highlights the importance of integrating thyroid function assessment into the management of geriatric syndromes, and encourages clinicians to pay closer attention to thyroid health in this population.

Some potential limitations of this study should be acknowledged. First, this is a single-center cross-sectional study aimed at exploring the potential mechanisms underlying the association between FT3/FT4 and frailty. Due to its cross-sectional nature, it is not possible to establish a causal relationship between the two. In addition, the external validation data were obtained from a single center, which may limit the generalizability of the results. Further research is warranted to explore the underlying molecular mechanisms and to elucidate the underlying molecular mechanisms and to validate these findings. This study is limited by the use of age-invariant TSH reference ranges, which may underestimate thyroid dysfunction prevalence and its association with frailty in older populations; future studies should validate findings using age-stratified thresholds.

## Conclusion

In conclusion, our study reveals a significant relationship between frailty and the ratio of FT3 to FT4. In addition, we constructed and validated a novel nomogram for assessing frailty in the older peolpe. The model performed well. The prediction model will be valuable in facilitating the screening and prediction of the risk of developing frailty in the older adults by community health organizations and clinical healthcare professionals.

## Supplementary Information

Below is the link to the electronic supplementary material.

Supplementary file1 (DOCX 310 KB)

## Data Availability

The datasets produced during the current study can be obtained from the corresponding author upon reasonable request.
