# Supplementary material for: Development and validation of a predictive nomogram for frailty based on thyroid function in older adults
Source: Eur Geriatr Med. 2025 Jun 17;16(5):1861–70. doi: 10.1007/s41999-025-01247-3 (PMC12528339; doi:10.1007/s41999-025-01247-3)
Supplement: Supplementary file 1 — Supplementary file1 (DOCX 310 KB) [file 41999_2025_1247_MOESM1_ESM.docx]

***
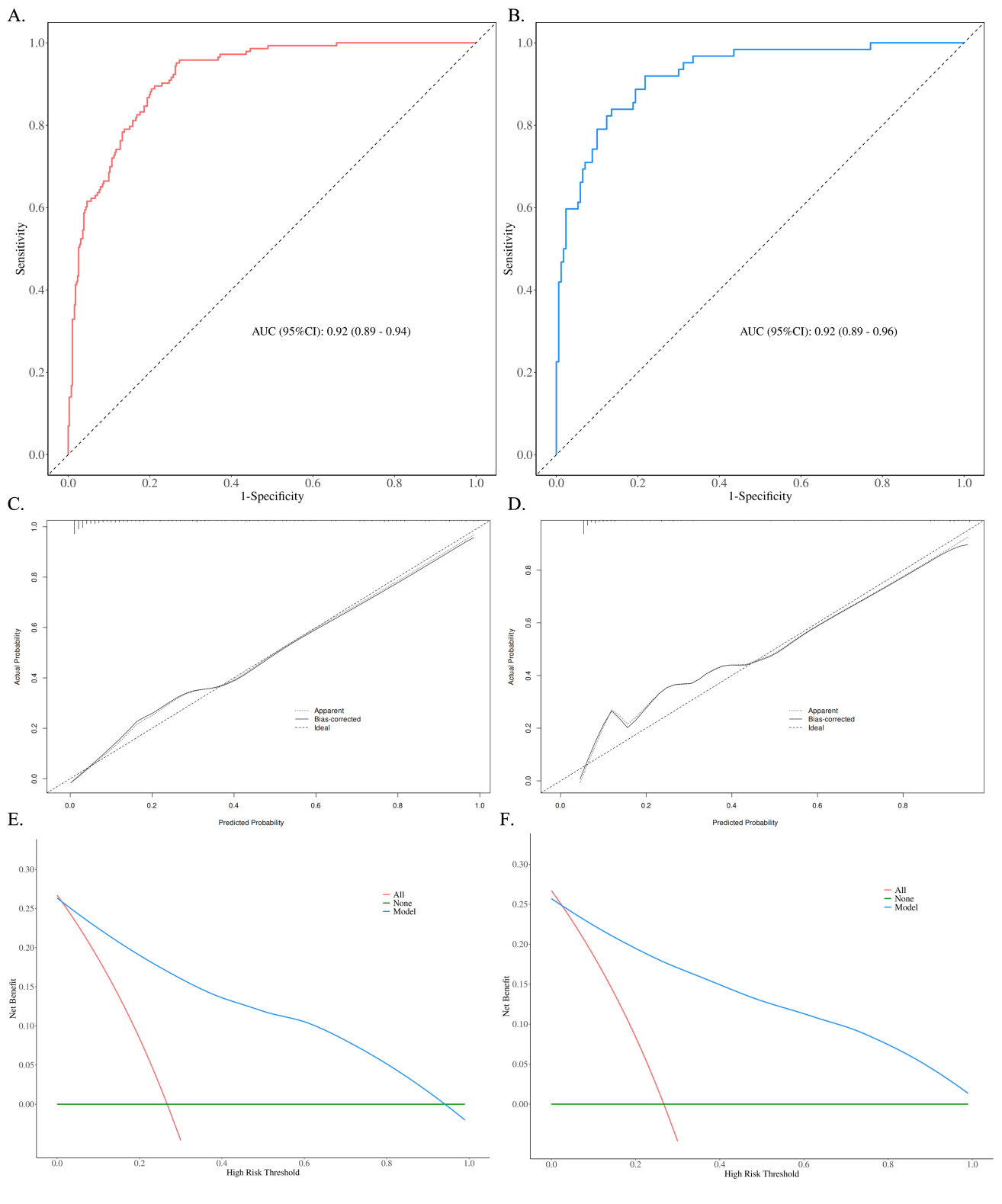
***

***Supplementary Figure 1.*** (A) The ROC for the training set; (B) The ROC for the validation set. (C) The calibration curve for the training set; (D) The calibration curve for the validation set. (E) The DCA for the training set; (F) The DCA for the validation set.

***
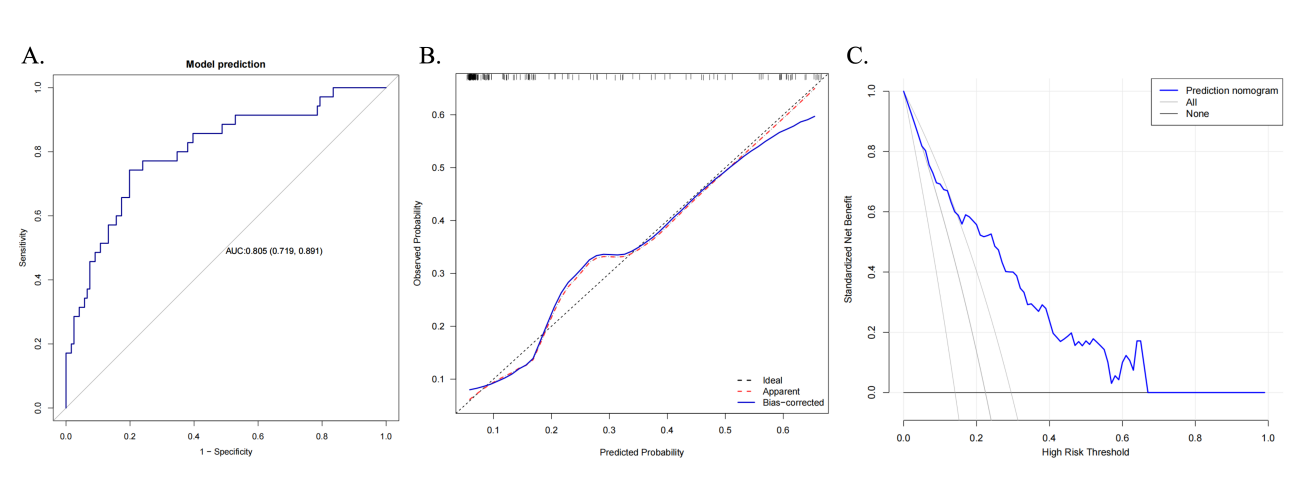
***

***Supplementary Figure 2.*** (A) The ROC for the external validation set; (B) The calibration curve for the external validation set; (C) The DCA for the external validation set.

| Supplementary Table 1.Association Between FT3/FT4 Ratio and Frailty Stratified by Nutritional Status (MNA-SF Categories) | | | |
| --- | --- | --- | --- |
| Nutritional Status | Sample Size | FT3/FT4 (OR, 95% CI) | p-value |
| Normal group | 523 | 0.05 (0.01, 0.24) | < 0.001 |
| Abnormal group (At-risk + malnutritionr) | 244 | 0.04 (0.01, 0.34) | < 0.05 |
| Interaction p-value | - | 0.19 | - |

| Supplementary Table 2 Relationship between FT3/FT4 and frailty in different models | | | |
| --- | --- | --- | --- |
| Variable | model 1(OR, 95%CI, p) | model 2 (OR, 95%CI, p) | model 3 (OR, 95%CI, p) |
| FT3/FT4 (quartile) | |  |  |
| Q1 | Reference | Reference | Reference |
| Q2 | 0.39 (0.26,0.60) <0.001 | 0.63 (0.40,0.99) <0.05 | 0.65 (0.36,1.17) >0.05 |
| Q3 | 0.21 (0.13,0.32) <0.001 | 0.44 (0.26,0.74) <0.05 | 0.77 (0.39,1.49) >0.05 |
| Q4 | 0.02 (0.01,0.06) <0.001 | 0.05 (0.02,0.14) <0.001 | 0.04 (0.01,0.13) <0.001 |

Crude model: we did not adjust other covariants
Minimally adjusted model: adjusted age and gender
Fully adjusted model: adjusted age, gender, BMI, smoking, GRNI, grip,HbA1c,WBC,RBC, HGB,CHOL,TG, HDL, LDL, UA, BUN, Cr, AST,ALT,T3,T4,FT3,FT4,TSH

| Supplementary Table 3 Association Between FT3/FT4 Ratio and Frailty Stratified by Nutritional Status (GRNI) | | | |
| --- | --- | --- | --- |
| Variable | Normal group(OR, 95%CI, p) | Mild risk group (OR, 95%CI, p) | Moderate-severe risk group(OR, 95%CI, p) |
| FT3/FT4 (quartile) | |  |  |
| Q1 | Reference | Reference | Reference |
| Q2 | 0.42 (0.07,2.46) >0.05 | 1.17 (0.49,2.80) >0.05 | 0.44 (0.18,1.04) >0.05 |
| Q3 | 0.85 (0.17,4.16) >0.05 | 1.52 (0.55,4.21) >0.05 | 0.45 (0.15,1.33) >0.05 |
| Q4 | 0.06 (0.01,0.52) <0.05 | 0.13 (0.02,0.73) <0.05 | 0.01 (0.01,1.13) >0.05 |
